# Epigenetic adaptation drives monocyte differentiation into microglia-like cells upon engraftment into the retina

**DOI:** 10.1101/2024.09.09.612126

**Published:** 2024-09-14

**Authors:** Jie Liu, Fengyang Lei, Bin Yan, Naiwen Cui, Jyoti Sharma, Victor Correa, Lara Roach, Savvas Nicolaou, Kristen Pitts, James Chodosh, Daniel E. Maidana, Demetrios Vavvas, Milica A Margeta, Huidan Zhang, David Weitz, Raul Mostoslavsky, Eleftherios I. Paschalis

**Affiliations:** a. Department of Ophthalmology, Massachusetts Eye and Ear, Harvard Medical School, Boston, MA 02114, USA; b. Department of Ophthalmology, The Second Xiangya Hospital of Central South University, 139 Middle Renmin Road, Changsha, Hunan, 410011, China; c. School of Engineering and Applied Sciences (SEAS), Harvard University, Cambridge, MA, USA.; d. Center for Regenerative Medicine, Massachusetts General Hospital, Harvard Medical School, Boston, MA 02114, USA; e. Department of Ophthalmology and Visual Sciences, University of New Mexico School of Medicine, Albuquerque, NM 87131, USA; f. Department of Ophthalmology and Visual Sciences, Illinois Eye and Ear Infirmary, University of Illinois at Chicago, Chicago, IL

**Keywords:** microglia, marker, macrophages, monocytes, retina, Biological Science/Immunology and Inflammation

## Abstract

The identification of specific markers for microglia has been a long-standing challenge. Recently, markers such as P2ry12, TMEM119, and Fcrls have been proposed as microglia-specific and widely used to explore microglial functions within various central nervous system (CNS) contexts. The specificity of these markers was based on the assumption that circulating monocytes retain their distinct signatures even after infiltrating the CNS. However, recent findings reveal that infiltrating monocytes can adopt microglia-like characteristics while maintaining a pro-inflammatory profile upon permanent engraftment in the CNS.In this study, we utilize bone marrow chimeras, single-cell RNA sequencing, ATAC-seq, flow cytometry, and immunohistochemistry to demonstrate that engrafted monocytes acquire expression of established microglia markers—P2ry12, TMEM119, Fcrls—and the pan-myeloid marker Iba1, which has been commonly mischaracterized as microglia-specific. These changes are accompanied by alterations in chromatin accessibility and shifts in chromatin binding motifs that are indicative of microglial identity. Moreover, we show that engrafted monocytes dynamically regulate the expression of CX3CR1, CCR2, Ly6C, and transcription factors PU.1, CTCF, RUNX, AP-1, CEBP, and IRF2, all of which are crucial for shaping microglial identity. This study is the first to illustrate that engrafted monocytes in the retina undergo both epigenetic and transcriptional changes, enabling them to express microglia-like signatures. These findings highlight the need for future research to account for these changes when assessing the roles of monocytes and microglia in CNS pathology.

## Significance Statement

Monocytes can express putative microglia markers P2ry12, TMEM119, and Fcrls upon engraftment into the retina. Given their involvement in neuroinflammation, it is crucial to consider this overlap when utilizing these markers for experimental analysis.

## Introduction

Microglia and infiltrating peripheral monocytes play a central role in central nervous system (CNS) pathology and have become therapeutic targets in recent studies [1–5]. Given that these immune cell populations have overlapping biological functions, the use of markers for their differentiation often results in the inadvertent mislabeling, thereby compromising interpretation of relevant experimental data. More recently, P2ry12, TMEM119, and Fcrls were proposed as microglia “specific” markers and subsequently used in various studies[6, 7]. The specificity of these markers was originally assessed by comparing their expression in blood, spleen, or infiltrating monocytes against that of CNS-resident microglia. It was presumed that infiltrating monocytes would maintain their distinct characteristics following infiltration into the CNS and preserve these features for the long-term after engraftment into the tissue [6, 7].

However, recent findings indicate that infiltrating monocytes undergo persistent phenotypic changes upon engraftment into the CNS [2, 8]. These changes not only make them morphometrically similar to microglia but also allow them to permanently engraft into the tissue and live for the long-term, while retaining a pro-inflammatory that that can ultimately promote disease [2]. In previous studies, we identified “infiltrating monocytes” as ameboid, CCR2^hi^ CX3CR1^lo^ cells during the early infiltration phase (0–3 days), while engrafted monocytes were classified as ramified, CX3CR1^hi^ CCR2^lo^ cells typically migrating into distinct microglia strata [2].

In this study, we explore the ability of peripheral monocytes to express the putative microglia markers P2ry12, TMEM119, and Fcrls upon engraftment into the retina, and we investigate the mechanisms driving this phenotypic switch. We demonstrate that shortly after engraftment, monocytes begin expressing P2ry12, TMEM119, and Fcrls, while simultaneously undergoing dynamic changes in chromatin accessibility, binding motifs, and their transcriptome. This context-dependent adaptation allows engrafted monocytes not only to express microglia markers but also to modulate the expression of conventional monocyte/macrophage markers such as CX3CR1, CCR2, Ly6C, and key transcription factors including PU.1, CTCF, RUNX, AP-1, CEBP, and IRF2, which are crucial for reshaping their identity towards a microglia-like state.

This study highlights the significant plasticity of monocytes, showing that these cells can rapidly acquire microglia-like signatures upon engraftment into the retina. Distinguishing between embryonic microglia and monocyte-derived “microglia” is essential for understanding the distinct roles and functions of these two immune cell populations.

## Results

### Single-cell RNAseq reveals a transcriptional shift of engrafted monocytes towards a microglia signature.

**Figure 1.**
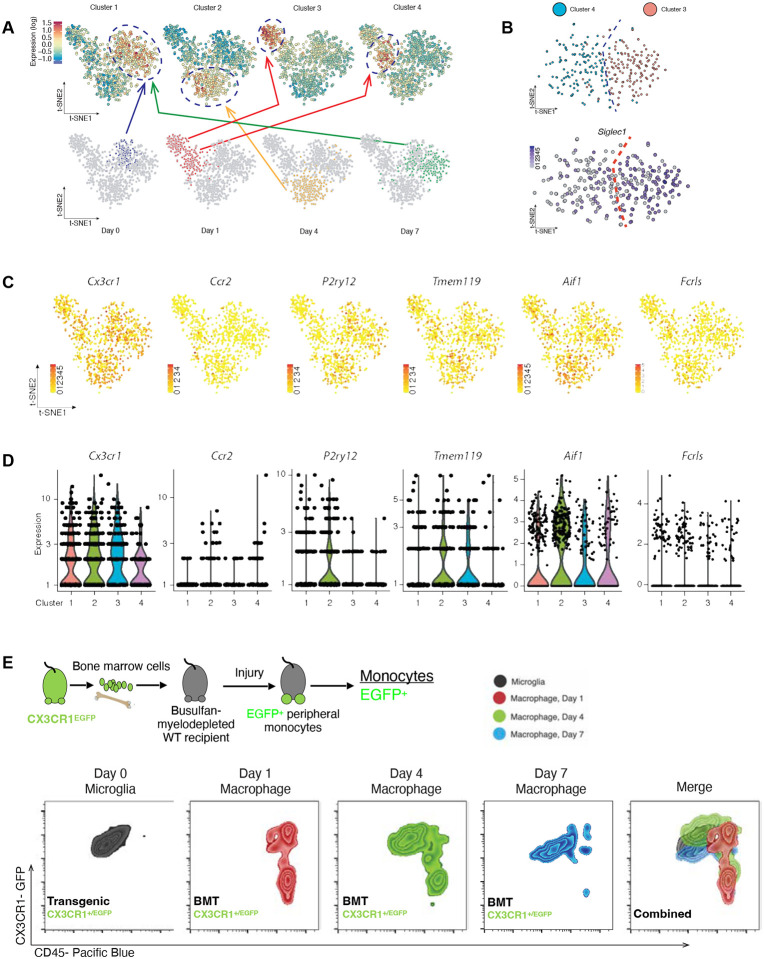
Single-cell RNAseq analysis for retinal CD45^+^CD11b^+^ cells 0, 1, 4 and 7 days after ocular injury. **(A)** Principal component analysis shows the existence of 4 clusters with distinct transcriptional profiles assigned to the day of cell retrieval. CD45^+^CD11b^+^ cells undergo extensive changes in their transcriptome during retinal engraftment and by day 7 acquire an identical signature to naive microglia. **(B)** Singlec1^+^ gene is expressed in clusters 3 and 4, both assigned to day 1, suggestive of the presence of monocytes within the microglia sample. **(C)** tSNE analysis of CX3CR1, CCR2, P2ry12m Tmem119, Aif1, and Fcrls expression in cluster 4 and **(D)** graphical representation of the expression of microglia markers P2ry12, Tmem119, Aif1, and Fcrls in the 4 clusters, including those representatives of microglia and monocytes signatures. **(E)** Flow cytometric analysis of the expression of CD45 marker in retinal CX3CR1^+^ cells before and 1, 4, and 7 days after injury. As reference we used a CX3CR1^+/EFGP^ mouse, stained with CD45 markers. Double positive CD45^+^ CX3CR1^+^ cells represent only microglia, since naïve eyes do not have infiltration of monocytes[2]. To map infiltrating/engrafting monocytes, we used a CX3CR1^+/EGFP^ bone marrow chimera. CX3CR1^+^ CD45^+^ infiltrating monocytes gradually transitioned their CD45 expression towards the expression of naïve microglia at 7 days.

Isolated CD45^+^ CD11b^+^ cells from the retina of wild-type mice were subjected to single-cell RNAseq prior to and 1, 4 and 7 days after ocular alkali injury. We previously showed that alkali injury to the cornea causes prompt infiltration of CCR2^+^ monocytes and their subsequent engraftment into the retinal tissue[2, 9]. Using t-SNE we analyzed 954 cell/sample and identified 4 clusters with unique transcriptional profiles (Fig. 1 A). Cluster 1 corresponded to yolk sack-derived native microglia, since prior to injury, peripheral monocytes do not infiltrate the retina[2–4, 9, 10]. One day after injury, monocytes and microglia formed two separate clusters; cluster 3 had high expression of Siglec1 gene, a monocyte marker [11–13] while cluster 4 had low expression, most indicative of microglia (Fig. 1 B). At 4 days, microglia and monocytes formed a single cluster 2, suggestive of transcriptional overlap and at day 7, both cell populations were contained within cluster 1 (yolk-sac derived naïve microglia) (Fig. 1 A), demonstrating a dynamic shift towards microglia signature. Gene expression analysis revealed the uniform expression of classical macrophage marker CX3CR1 in all clusters and predominant expression of CCR2 monocyte marker in cluster 2 (day 1) and 4 (day 4), (Fig. 1 C, D). P2ry12 was highly expressed in cluster 1 (day 0 and 7) and 2 (day 4), TMEM119 and Fcrls in all clusters (Fig. 1 C, D), and Aif1 (gene of IBA1) in cluster 2 (day 4). P2ry12 expression exhibited temporal regulation in monocytes, with lack of expression in clusters 3 and 4 – both representing early-stage infiltration (Day 1) (Fig. 1 C, D). Next, a CX3CR1^+/EGFP^ bone marrow chimera model was employed, as previously described[2, 4, 14], to differentiate the two immune cell populations (monocytes/microglia) using flow cytometry. Seven days after injury, engrafted CX3CR1^+^ cells had adopted a similar CD45 expression as embryonic microglia, corroborating scRNAseq results of converging signature (Fig. 1E). The results of scRNAseq were further confirmed using qPCR on bone marrow chimeras, which showed that monocytes acquire expression of P2ry12, TMEM119, Fcrls, and Iba1 genes within 45 days of engraftment (Fig. S1). In contrast, embryonic microglia retained their expression in naïve and injured eyes (Fig. S1).

### Monocytes acquire de-novo expression of P2ry12, Fcrls, and TMEM119 in the protein level upon engraftment into the retina.

**Figure 2.**
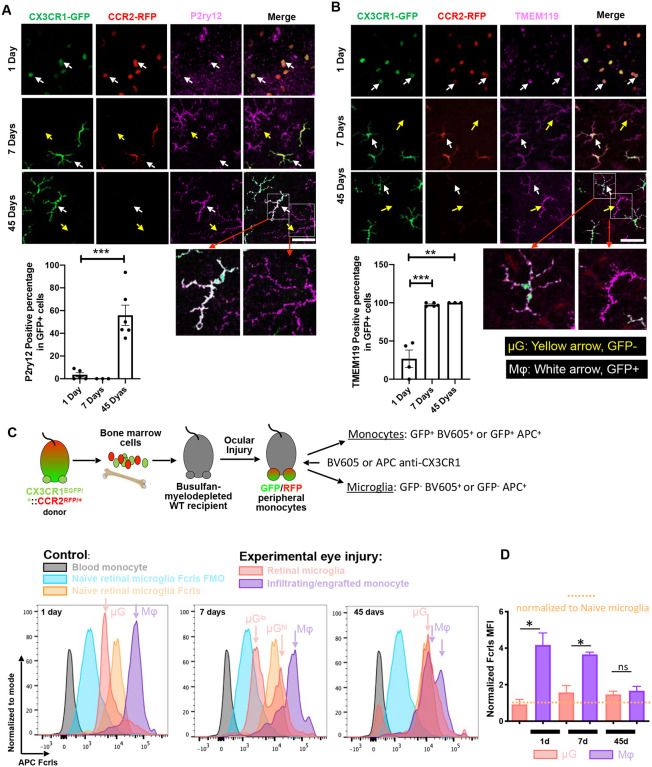
Protein expression of P2ry12, TMEM119 and FCRLS by engrafted monocytes. CX3CR1^+/GFP^::CCR2^+/RFP^ bone marrow chimeras were used to differentiate engrafted monocytes from embryonic microglia, followed by immunostaining and flow cytometry to assess expression of P2ry12, TMEM119, and FCRLS proteins. **(A)** P2ry12 is not expressed at day 1 and day 7 after monocyte infiltration into the retina, however 55% of GFP^+^ engrafted monocytes showed positive expression of P2ry12 at day 45. ***, *P* < *0.001*. **(B)** Twenty-five percent of GFP^+^ peripheral monocytes (white arrow) expressed TMEM119 at day 1. By day 7, all GFP^+^ engrafted monocytes are TMEM119^+^. TMEM119 expression in engrafted monocytes is retained at day 45.**, *P* < *0.01*; ***, *P* < *0.001*. **(C-D)** A CX3CR1^+/GFP^::CCR2^+/RFP^ bone marrow chimera was employed to assess Fcrls expression in monocytes and microglia by flow cytometry. BMT CX3CR1^+^ cells were labeled with a conjugated antibody against CX3CR1 (BV605 or APC) which allowed differentiation between embryonic microglia (BV605^+^GFP^−^ or APC^+^GFP^−^) and engrafted monocytes (BV605^+^GFP^+^ or APC^+^GFP^+^). Blood monocytes had no Fcrls expression (grey). One day after infiltration, BV605^+^GFP^+^ peripheral monocytes (purple) acquired strong Fcrls expression, which was comparable to naïve embryonic microglia. Fcrls expression was retained by engrafted monocytes sustained at day 7. At day 45, Fcrls expression was similar to embryonic microglia in the same injured tissue or to naïve microglia. *, *P* < *0.05. MFI: Median Fluorescence Intensity*. Yellow arrows indicate GFP^−^ microglia and white arrows GFP^+^ engrafted monocytes. (A,B) *Scare bar = 50 μm*.

To determine whether the observed transcriptional changes in P2ry12, TMEM119, Fcrls, and IBA1 expression translate to the protein level in engrafted monocytes, we performed dual flow cytometry and immunohistochemistry analysis using a CX3CR1^+/GFP^::CCR2^+/RFP^ bone marrow chimera[2, 4, 14]. We first studied the CX3CR1^+/GFP^::CCR2^+/RFP^ double transgenic mouse to confirm that circulating monocytes (before engraftment) do not express P2ry12, TMEM119, or Iba1 in the protein level (Fig. S2). We also confirmed that circulating monocytes express MHC-II (Fig. S2). Using flow sorting of CD45^+^ CD11b^+^ CX3CR1^+^ blood cells from naïve mice, we confirmed the absence of Fcrls protein in circulating monocytes (Fig. 2 C).

One- and seven-days post-injury, GFP^+^ engrafted monocytes had no detectable expression of P2ry12 (white arrow), (Fig. 2 A). By day 14, engrafted monocytes have transformed to ramified monocytes, acquired expression of P2ry12 and appearing morphometrically similar to microglia (Fig. S3). At 45 days, engrafted monocytes had heterogeneous expression of P2ry12 with either positive or negative P2ry12 protein expression with approximately 55% of GFP^+^ engrafted monocyte being P2ry12 positive (Fig. 2 A). The percentage of P2ry12 positive cell significantly increased compared to 1-day post-injury (Fig. 2 A). In contrast, embryonic microglia (GFP^−^) retained robust expression of P2ry12 protein (yellow arrow) throughout the study period (Fig. 2 A).

Likewise, 25% of GFP^+^ peripheral monocytes (white arrow) expressed TMEM119 at 1-day post-injury (Fig. 2 B). By day 7, all GFP^+^ engrafted monocytes were TMEM119^+^, and this expression was sustained on day 45 of engraftment (Fig. 2 B). The percentage of TMEM119 positive cell significantly increased compared to 1-day post-injury (Fig. 2 B). GFP^−^ embryonic microglia (yellow arrow) displayed robust expression of TMEM119 protein throughout the study period (Fig 2 B).

Expression of Fcrls was assessed in CX3CR1^+/EGFP^::CCR2^+/RFP^ bone marrow chimeras by flow cytometry. First, CX3CR1^+^ cells were labeled with a conjugated antibody against CX3CR1 (BV605 or APC). BV605^+^GFP^−^ or APC^+^ GFP^−^ cells were classified as embryonic microglia, and BV605^+^GFP^+^ or APC+ GFP^+^ were classified as infiltrating peripheral monocytes (Fig 2 C). Consistent with the above-mentioned findings, Fcrls was not expressed in blood monocytes (grey)[6], though 1 day after infiltration into the retina. BV605^+^GFP^+^ peripheral monocytes (purple) acquired higher expression of Fcrls as compared to embryonic microglia at baseline (Fig 2. C, D). In parallel, microglia reduced Fcrls expression below their baseline level (Fig. 2 C, D). Peripheral monocytes sustained high Fcrls expression at 7 days, as compared to baseline microglia. By 45 days after injury, this expression was not significantly different between embryonic microglia and engrafted monocytes (Fig. 2 C, D). Fcrls expression waned in infiltrating monocytes and increased in microglia after injury, reaching similar levels at 45 days between these two immune populations, which was equivalent to that of naive microglia at baseline (Fig. 2 D).

IBA1 was expressed in GFP^+^ monocytes (white arrow) already 1 day after engraftment into the retina, with 85% of the cells being IBA1^+^. By day 7, all GFP^+^ engrafted monocytes were expressing IBA1 and retained that expression until day 45 (Fig. S4). GFP^−^ embryonic microglia (yellow arrow) displayed robust expression of IBA1 throughout the study period (Fig S4).

### Monocytes undergo chromatin accessibility changes upon engraftment into the retina.

Perturbances in tissue homeostasis often result in gene regulation and chromatin accessibility changes with subsequent transcription/translation reshaping of key proteins in monocytes [15–17]. Here, we employed the Assay for Transposase-Accessible Chromatin with sequencing (ATAC-seq) to study the effect of monocyte engraftment in gene accessibility for putative microglia markers P2ry12, Fcrls, TMEM119, and Iba1 in flow-sorted microglia and peripheral monocytes using a bone marrow chimera model (Fig. 2 A).

**Figure 3.**
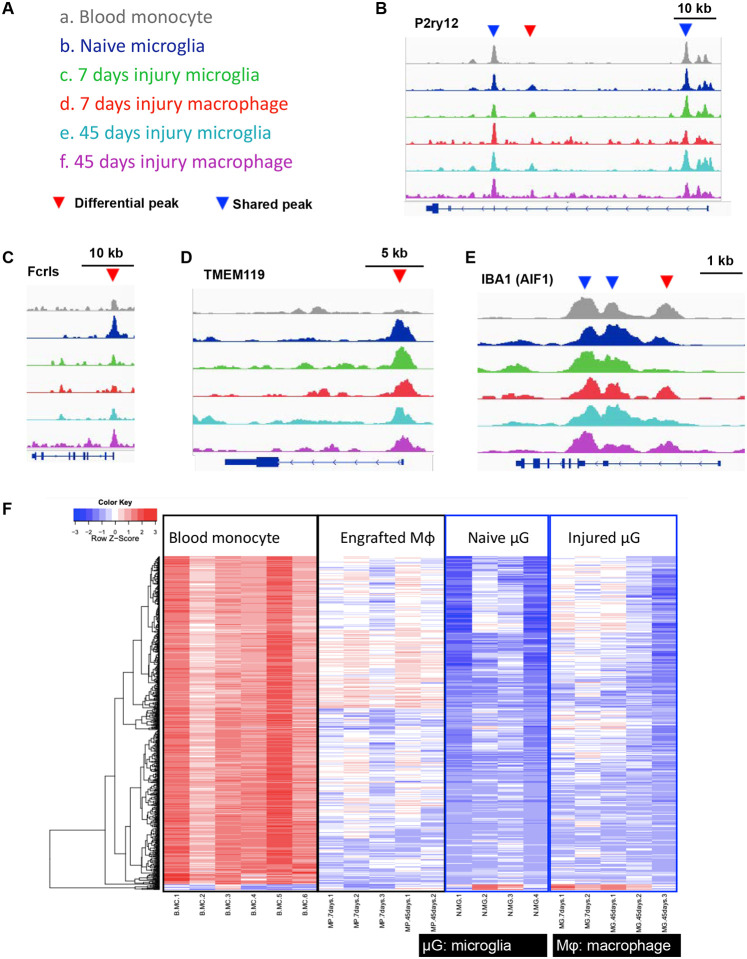
ATAC-seq analysis to assess chromatin accessibility for P2ry12, FCRLS, Aif1 (IBA1), and TMEM119 genes in engrafted monocytes and microglia. **(A)** Color coding of analyzed groups. **(B)** P2ry12 gene contains 3 open chromatin peaks, 2 of the peaks (blue arrowhead) are similar between the groups, but the 3rd peak is present in microglia (red arrowhead) but not in circulating monocytes. Upon engraftment into the retina, monocytes acquire the 3rd peak (red arrowhead) which is retained throughout the study period (45 days). **(C)** Open chromatin peaks for Fcrls gene appear similar between the groups, with differences only in the amplitude of the peaks at 45 days in microglia and engrafted monocytes which have higher peaks compared to circulating monocytes or monocytes during early engraftment into the retina (7 days). **(D)** Open chromatin peaks for Aif1 gene (IBA1) appear similar between the groups, although microglia appeared to abolish one peak (red arrowhead) at 7 and 45 days after the injury. **(E)** TMEM119 has only one open chromatin peak, which is present in microglia but not in circulating monocytes, but upon engraftment, peripheral monocytes acquire this distinct peak (red arrowhead). **(F)** Heat map analysis of consensus peaks, suggests that monocytes undergo significant open chromatin alterations upon engraftment into the retina which allow differentiation from circulating monocytes. Monocytes increase chromatin accessible for genes P2ry12, Tmem119, Fcrls, and Aif1 upon engraftment into the retina eventually acquiring a similar open chromatin signature to microglia.

In ATAC seq, Peak width refers to the horizontal extent of a peak in the ATAC-seq signal track. It represents the range over which chromatin accessibility is elevated. A wider peak indicates a broader region of accessible chromatin. Peak amplitude refers to the height or intensity of the peak in the ATAC-seq signal track. It represents the level of chromatin accessibility at the peak’s center. Higher amplitude suggests more frequent chromatin accessibility in that region. In the current study, circulating monocytes and naïve microglia were used as controls. Groups and color coding are listed in (Fig. 3 A). Engrafted monocytes showed similar open chromatin peaks to circulating monocytes (blue arrows) but also displayed new open chromatin peaks not previously present in circulating monocytes (red arrows) but present in native (embryonic) microglia. One of the peaks under the P2ry12 gene in naïve microglia (red arrow) was absent in circulating monocytes but acquired upon engraftment into the retina (Fig. 3 B). The Fcrls gene had similar open chromatin peak across all groups (Fig. 3 C), though naïve microglia had wider and higher peak as compared to circulating monocytes (blue peak compared to grey). After engraftment, the amplitude of this peak increased in monocytes and became similar to microglia at day 45 (blue peak compared to purple), (Fig. 3 C). Similarly, TMEM119 chromatin was not accessible in circulating monocytes, however, it became accessible after monocyte engraftment into the retina (red arrow), (Fig. 3 D). Lastly, AIF1 gene (IBA1) had similar open chromatin peaks among the groups, corroborating the above transcriptional and protein findings showing gain of IBA1 expression by monocytes after engraftment into the retina (Fig. 3 E).

Differential chromatin accessibility peaks were identified by comparing blood monocytes to naive microglia or engrafted monocytes at 7 or 45 days. Heatmap analysis displayed significant alterations in chromatin accessibility between the samples (Fig. 3 F) with more than 3000 differential chromatin accessibility peaks identified between peripheral and engrafted monocytes at 7 days. These peak profiles remained stable at 45 days. Similarities in chromatin accessibility between engrafted monocytes and microglia confirm our hypothesis that gradual transition of chromatin state in engrafted monocytes facilitates their adaptation into the retina (Fig. 3 F).

**Figure 4.**
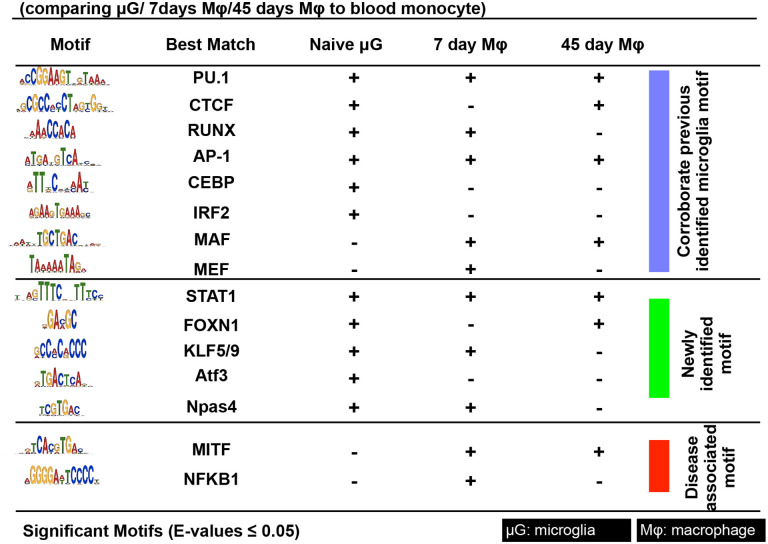
ATAC-seq motif analysis for discovery of putative transcription factors regulating monocyte engraftment. Motif analysis of differential open chromatin peaks identified by comparing naive microglia, retinal engrafted monocyte (7 and 45 days), and circulating monocytes. Blue section contains motifs assigned to transcription factors previously identified in human and mouse microglia, such as PU.1 (most common), CTCF, IRF, RUNX, MEF2, C/EBP, AP-1, MAF, and MEF. Green section contains enriched motifs assigned to novel transcription factors, such as STAT1, FOXN1, KLFs, ATF3, and Npas4. Red section contains previously reported disease associated motifs, such as MITF and NFKB1. Analysis of naive microglia identifies multiple reported factors but not MAF and MEF, which are identified only in engrafted monocytes. Engrafted monocyte at 7 and 45 days contain highly enriched motifs assigned to the above-mentioned transcription factors CEBP, IRF2, and ATF3. Disease-associated motifs assigned to MITF and NFKB1 are identified in engrafted monocytes but not in microglia. *E-value* <*0.05 for statistically significant motifs*.

Motif analysis of differential chromatin accessibility peaks was performed to identify the most highly enriched transcription factor recognition motifs between circulating monocyte and naive microglia or engrafted monocytes at 7 or 45 days. We identified motifs assigned to PU.1 (most dominant), CTCF, IRF, RUNX, MEF2, C/EBP, AP-1 in naive microglia, corroborating previous findings [18] apart from motifs for MAF and MEF which were previously shown only in microglia. In addition, we found other enriched motifs, including STAT1, FOXN1, KLFs, ATF3, and Npas4, which have been reported to be associated with microglia functions of polarization, cytokine production, suppression of inflammation and phagocytosis (22–26) (Fig. 4). Engrafted monocytes had highly enriched motifs assigned to the above-mentioned transcription factors, which we summarized in Fig. 4. In addition, engrafted monocytes exhibited enriched motifs assigned to MITF and NFKB1, known to be responsible for disease-associated transcriptional signatures [19] and promotion of inflammation, respectively (Fig. 4). ATAC-seq analysis suggests that monocytes undergo dynamic open chromatin accessibility changes upon engraftment into the retina, which affects their phenotype and allows them to acquire putative microglia signatures, although they remain somewhat functionally distinct in terms of promoting retinal neurodegeneration [2].

### Monocytes undergo extensive changes in protein expression upon engraftment into the retina.

**Figure 5.**
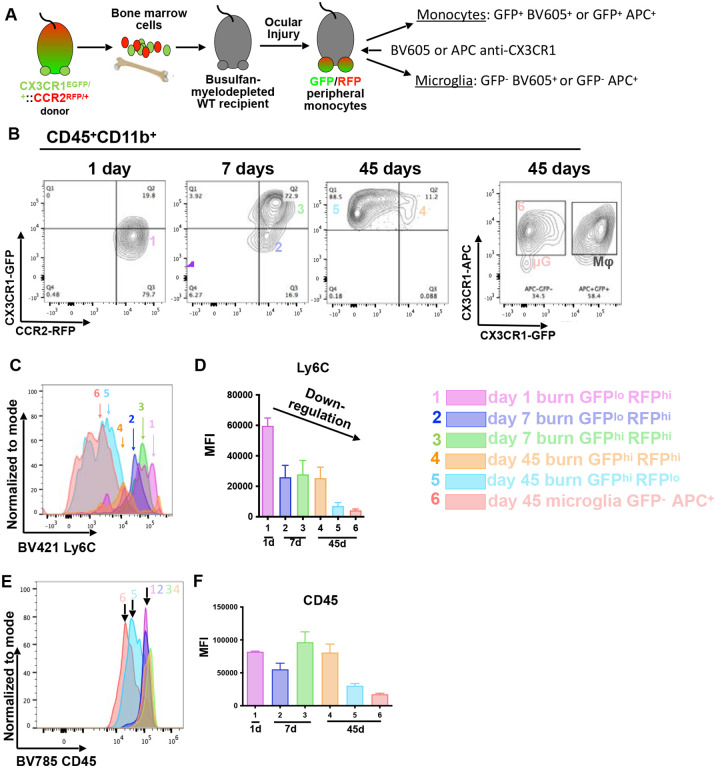
Expression of conventional markers by microglia and engrafted monocytes. **(A)** Development of a CX3CR1^+/GFP^::CCR2^+/RFP^ bone marrow chimera model to differentiate microglia from peripheral monocytes using flow cytometry and a gating strategy as follows: microglia: GFP-negative CX3CR1/BV605+positive or GFP-negative CX3CR1/APC+positive, engrafted monocytes: GFP+positiveCX3CR1/BV605+positive or GFP+positiveCX3CR1/APC+positive. (B) Peripheral monocyte/macrophages repress CCR2 expression and enhance CX3CR1 expression during engraftment into the retina. Five distinct maturation phases of monocytes after engraftment are identified (Groups 1–5). A separate group of CCR^−negative^CX3CR1^−negative^ cells representing naive microglia (Group 6; μG) is retained as a population throughout the study period (45 days). **(C-D)** Engrafted monocytes have increase expression of Ly6C at day 1 of infiltration, which is gradually suppressed during engraftment. At 45 days, the majority of engrafted monocytes (Group 5) have similar Ly6G expression as microglia (Group 6). **(E-F)** Engrafted monocytes exhibit sustained expression of CD45 at days 1 and 7 followed by repression in subpopulations of these cells (Group 5), and at day 45 reaching equal levels compared to retinal microglia (Group 6).

Further characterization of the protein expression changes in engrafted monocytes was performed using established markers Ly6C, CD45, and MHC-II in CX3CR1^+/GFP^::CCR2^+/RFP^ bone marrow chimeras (Fig. 5 A). Infiltrating monocytes (CD45^+^ CD11b^+^) were CCR2^high^ CX3CR1^low^ at day 1 (Group 1), (Fig. 5 B), and by day 7 were either CCR2^high^ CX3CR1^low^ (Group 2) or CCR2^+^ CX3CR1^hi^ (Group 3). At 45 days, engrafted monocytes were CCR2^low/−^ CX3CR1^high^ (Group 5) with a small subpopulation being CCR2^high^CX3CR1^high^ (Group 4), (Fig. 5 B). CX3CR1^−GFP^ -negative CX3CR1^−APC^ -positive microglia were isolated 45 days after injury as controls (flow cytometry staining Group 6), (Fig. 5 B).

**Figure 6.**
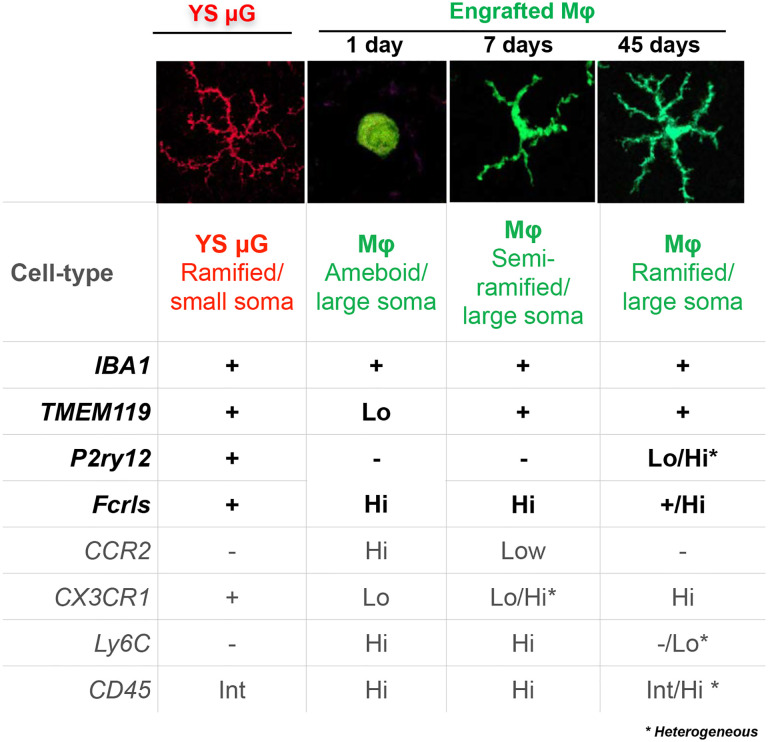
Summary of the microglia and monocyte markers changes Monocyte transition from highly amoeboid to highly ramified cells during engraftment into the retina, becoming morphometrically identical and indistinguishable from retinal microglia. These changes are accompanied by suppression of monocyte markers CCR2, Ly6C, and CD45, and upregulation of the tissue-resident macrophage marker CX3CR1^+/GFP^ and microglia markers IBA1, TMEM119, P2ry12, and FCRLS. P2ry12 appears to be conditionally specific to microglia during early infiltration of monocytes) and to a subpopulation of engrafted monocytes.

CCR2^high^ monocytes displayed high expression of Ly6C at day 1, which gradually declined as cells transitioned to CCR2^low/-^ CX3CR1^high^ during engraftment (Group 5), (Fig. 5 C, D). In contrast, engrafted monocytes that retained CCR2^high^CX3CR1^high^ expression also displayed sustained elevated expression of Ly6C throughout the study period (Group 4) (Fig. 5 C, D). CD45 expression was similar to CCR2 expression; both markers were repressed in monocytes during engraftment (Fig 5 E, F). The expression patterns of P2ry12, TMEM119, Fclrs, IBA1, CCR2, CX3CR1, Ly6C, and CD45, with monocyte and microglia morphometric characteristics[20] are summarized in (Fig. 6).

## Discussion

The exact role of microglia in central nervous system (CNS) pathology remains a subject of ongoing scientific debate. Numerous studies have reported both protective and deleterious roles for microglia across various CNS diseases, adding complexity to our understanding of their function [21–25]. This debate is compounded by technical limitations and the lack of specificity in microglia/monocyte markers, making it difficult to distinguish between these two immune cell populations [21, 22]. The challenge is further heightened by the ability of peripheral monocytes to infiltrate the CNS during disease, engraft permanently, and adopt a microglia-like morphology, complicating the identification of these cells[2]. Fate mapping studies have revealed that engrafted monocytes tend to exhibit a more pro-inflammatory phenotype compared to resident microglia[2, 26], emphasizing the critical need for accurate differentiation between these cell types in research studies. However, the absence of definitive markers has exacerbated the complexity of this issue.

Recently, markers such as P2ry12, FCRLS, TMEM119, and Iba1 were proposed as microglia-specific and have been rapidly adopted by the research community[6, 7, 27, 28]. Despite their widespread use, uncertainties persist regarding the specificity of these markers, particularly in the context of monocyte engraftment into CNS tissue. In this study, we utilized single-cell RNA sequencing, ATAC sequencing, and protein analysis to evaluate the expression of P2ry12, FCRLS, TMEM119, and Iba1 in engrafted monocytes within the retina, and to explore the chromatin accessibility changes that may contribute to the phenotypic switch observed in these cells.

TMEM119 has been highlighted as a promising microglia-specific marker, particularly in models of optic nerve injury, where it was shown to differentiate microglia from infiltrating CCR2^RFP/+^ peripheral monocytes [7]. However, it has also been demonstrated that CCR2^−^ TMEM119^+^ peripheral monocytes can populate the injured optic nerve and contribute to the inflammatory environment [2, 4]. Our findings confirm that, in addition to microglia, CCR2+ CX3CR1^+^ engrafted monocytes express TMEM119 as early as one day post-injury, with sustained expression at 45 days. These results align with previous reports of TMEM119 expression in other tissues [29, 30], raising important questions about the interpretation of data from past studies.

Additionally, P2ry12 and Fcrls have emerged as potential microglia-specific markers, but their specificity was initially assessed by comparing CNS-derived CD45^lo^ CD11b^+^ microglia with splenic CD11b+ Ly6C+ monocytes in naïve adult mice[6]. This comparison did not account for engrafted monocytes. Additional studies using models of autoimmune encephalitis (EAE) suggested that P2ry12 and Fcrls were not expressed in infiltrating monocytes during early EAE onset[6]. However, our data show that both markers are indeed expressed by peripheral monocytes after engraftment into the retina. Fcrls is expressed as early as one day post-injury, with sustained expression, while P2ry12 is differentially expressed starting 14 days post-engraftment, with some monocytes retaining P2ry12 expression throughout the study period. These findings challenge the current understanding of these markers and suggest that previous studies may need reevaluation, particularly in the context of neurodegenerative diseases such as Alzheimer’s, where P2ry12-negative microglia have been reported surrounding Aβ plaques [31, 32]. Our data suggest that these cells could instead be engrafted monocytes, further complicating the interpretation of microglia-specific roles in such contexts.

Although Iba1 is a well-known pan-myeloid marker, it has been frequently misused as a microglia-specific in studies of tauopathy and multiple sclerosis [29, 32–38]. Using bone marrow chimeras, we confirmed that Iba1 is expressed in both microglia and engrafted monocytes, indicating that it should not be used to differentiate these immune cell populations. Similarly, conventional markers like CD45^lo^ and CD11c^lo^, previously proposed to distinguish microglia from peripheral monocytes/macrophages [39], proved inefficient in our study. Specifically, the CD45^hi^ CD11b^+^ signature, often used for differentiation, was inadequate outside the very acute phase of the experiment, as the majority of engrafted monocytes repressed CD45 expression within 45 days, reaching levels comparable to CD45^lo^ CD11b^+^ microglia, as shown in this study and by others [39, 40].

Mechanistically, the expression of P2ry12, FCRLS, TMEM119, and Iba1 in engrafted monocytes is associated with chromatin changes that enhance the accessibility of these genes. Our ATAC-seq analysis revealed that engrafted monocytes undergo significant chromatin accessibility changes, allowing them to acquire epigenetic signatures similar to those of microglia. Additionally, other established microglia genes, such as SPP1, C1qa, and Ms4a7, become accessible after monocyte engraftment, a feature not observed in circulating blood monocytes. Whether these changes in chromatin accessibility translate to transcriptional and protein-level expression requires further investigation [22, 40].

Understanding the molecular mechanisms that enable engrafted monocytes to acquire microglia signatures is crucial for advancing our knowledge of neuroglia remodeling. Previous studies have shown that transcription factors PU.1 (SPI-1) and Irf8 are essential for microgliogenesis, while Batf3 and Klf4 are not [41]. Our motif analysis between circulating and engrafted monocytes identified enriched transcription factor recognition motifs associated with PU.1, CTCF, IRF, RUNX, MEF2, C/EBP, AP-1 (JUN/FOSB/BATF3), and MAF, present in both human and mouse microglia [18]. This indicates a dynamic shift in the transcriptional network that supports the differentiation of engrafted monocytes into microglia. Interestingly, while SALL1 and SMAD4 have been implicated in microglia development and the expression of P2ry12 and TMEM119 [42], these motifs were not enriched in our dataset, suggesting that their role in monocyte identity transformation after engraftment may be less significant than previously thought. Future studies should further explore the biological roles of the transcription factors identified in our motif analysis.

There are limitations to this study that should be acknowledged. The use of a bone marrow chimera model was necessary to differentiate microglia from engrafted monocytes. While this model achieves stable chimerism and preserves blood-retinal barrier integrity, it requires myeloablation and conditioning, which may affect hematopoiesis. We previously showed that busulfan meylodepletion achieves stable chimerism and preserves blood-retinal barrier integrity[2]. A parabiosis model, although potentially more reliable, is limited by low-level chimerism [43]. Additionally, while we provide data on chromatin accessibility using ATAC-seq, further experiments employing techniques like CUT&RUN [44] or ChIP-seq [18] would be valuable in elucidating the epigenetic mechanisms governing gene expression in engrafted monocytes and microglia. Another limitation is the need to explore whether our findings are applicable to other CNS compartments, such as the brain, as marker expression may vary across different tissues and pathological contexts.

Despite demonstrating that infiltrating monocytes can acquire microglia signatures at the epigenetic, transcriptional, and protein levels, these cells remain distinct by expressing higher levels of inflammatory cytokines, such as TNF-α and IL-1β, compared to resident microglia [2]. Moreover, they exhibit enriched motifs associated with disease-associated and pro-inflammatory transcription factors MITF and NFKB1, respectively [19, 45]. Therefore, further analysis is required to fully delineate the functional differences between infiltrating monocytes and microglia in the diseased CNS.

In conclusion, the implementation of newly developed microglia markers requires careful validation to avoid misinterpretation of experimental data. While transgenic and lineage-tracing models offer short-term solutions [2, 8, 46, 47], the development of reliable protein markers are essential for long-term progress in the field. Until such markers are established, the scientific community must remain vigilant about potential pitfalls when interpreting both existing and new data.

## Materials and Methods

### Bone-Marrow Chimera Mouse Model.

All animal-based procedures were performed in accordance with the Association for Research in Vision and Ophthalmology Statement for the Use of Animals in Ophthalmic and Vision Research. This study was approved by the Animal Care Committee of the Massachusetts Eye and Ear Infirmary. Mice were bred in-house at the Massachusetts Eye and Ear Animal Facility and were used at the age of 6–12 wk.

A CX3CR1^+/GFP^::CCR2^+/RFP^ bone marrow transfer model was used to distinguish periphery infiltrated monocytes from CNS resident microglia [2]. Briefly, C57BL/6J mice (Recipient mice) were myelodepleted with three i.p. injections of busulfan (35 mg/kg; Sigma-Aldrich), an alkylating agent that depletes bone-marrow cells, 7, 5, and 3 d before BMT. CX3CR1^+/EGFP^::CCR2^+/RFP^ (donor mice) bone-marrow cells (5 × 10^6^ total bone-marrow cells) were transferred to the myelodepleted C57BL/6J mice through tail vein injection 1 month before corneal alkali burn. Bactrim (trimethoprim/sulfamethoxazole resuspended in 400 mL drinking water) was given ad libitum for 15 days after busulfan treatment.

Recipient mice C57BL/6J (stock no. 000664), and breeder mice including B6.129(Cg)-Ccr2tm2.1lfc/J mice (stock no. 017586) and B6.129PCx3cr1tm1Litt/J mice (stock no. 005582) were obtained from Jackson Laboratory. Donor mice CX3CR1^+/EGFP^::CCR2^+/RFP^ mice were generated by breeding male B6.129(Cg)-Ccr2tm2.1lfc/J mice with female B6.129PCx3cr1tm1Litt/J.

### Mouse Model of Alkali Burn.

One month after the CX3CR1^+/GFP^::CCR2^+/RFP^ bone marrow transfer model was established, corneal alkali chemical burns were performed according to our previous study [2]. In brief, mice were anesthetized using ketamine (60 mg/kg) and xylazine (6 mg/kg), and deep anesthesia was confirmed by toe pinch. A proparacaine hydrochloride USP 0.5% ophthalmic solution (Bausch and Lomb) was applied to the cornea and after 1 min was carefully dried with a Weck- Cel (Beaver Visitec International, Inc.). A 2-mm-diameter filter paper was soaked in 1 M sodium hydroxide (NaOH) solution for 10 s, dried of excess alkali, and applied onto the mouse cornea for 20 s. After the filter paper was removed, prompt irrigation with sterile saline was applied for 10 s. The mouse was then positioned laterally on a heating pad, and the eye was irrigated for another 15 min at low pressure using sterile saline. Ethiqa XR (buprenorphine) extended-release injectable suspension (3.25 mg/kg) (Covetrus North America, Cat: FP-001) was administered s.c. for pain management. A single drop of topical Polytrim antibiotic (polymyxin B/trimethoprim; Bausch & Lomb, Inc.) was administered after the irrigation. Mice were kept on the heating pad until fully awake.

### Flow Cytometry.

Flow cytometry was used to test for FCRLS, to investigate Ly6C and CD45 expression, and to sort cells for ScRNAseq and ATAC-seq. Infiltrated monocytes were gated as CD45^+^CD11b^+^GFP^+^ cells and microglia is CD45^+^CD11b^+^Cx3cr1^+^GFP^−^. Using the CX3CR1^+/GFP^::CCR2^+/RFP^ bone marrow transfer model, 1 day, 7 days and 1.5 months after corneal alkali burn injury, mouse retinas were collected and single cell suspensions prepared by papain digestion (Worthington Biochemical Corporation, Cat: LK003150). After digestion, cells were blocked with CD16/32 (clone: 2.4G2) and stained with primary antibodies. Antibody information can be found in Table 1. Samples were analyzed on a BD FACSAria^™^ III cell sorter and analyzed by FlowJo software.

### Single cell RNAseq and gene expression profiling:

To perform drop-based encapsulation, flow sorted CD45^+^CD11^+^ cells were encapsulated into micro droplets (≈50μm in diameter) using microfluidics (Fig. 6). The drops contain lysis buffer and RNase inhibitor to maximize efficiency. Barcoded hydrogel beads, with template-switching mastermix, were picoinjected into the droplets using high throughput microfluidic pico-injector. Once the cDNA was synthesized in-drop by captured mRNA, the drops (samples) were pooled and processed using Illumina HiSeq (deep sequencing) (Fig. 7). Cell identification was performed using the unique sequencing index as well as DNA barcode, during bioinformatic analysis. Library preparations of DNA for next generation sequencing were made according to Klein et al [25]. Paired-end sequencing (100bp) was performed with approximately 1,000 cells per sample on 1 lane of an Illumina HiSeq 2500. Reads were preprocessed and analyzed as described in the preliminary studies except that a custom reference transcriptome composed of the hg38 human reference transcriptome plus all BKV transcripts was used during Bowtie mapping. Gene counts werre normalized to the total number of mapped gene reads in each sample (RPM or reads per million). To confirm our results, we also tested other normalization methods such as DESeq2 [26]. Ingenuity pathway analysis (IPA^®^) from Qiagen was employed to understand the biological context of the single-cell RNAseq data and identify major pathways, regulatory networks, and causal relationships associated with the results.

### RNA isolation and Quantitative real-time PCR analysis

A bone marrow transfer model was used to distinguish periphery infiltrated monocyte and resident microglia. Naïve microglia cells and blood monocyte were collected from uninjured bone marrow transferred mice. Injured microglia and engrafted monocyte were collected from retinas 45 days after ocular injury in bone marrow transferred mice. Cells were directly collected into 1ml Trizol (Thermo Fisher, Cat: 15596026). RNA extraction was performed per standard assay recommendations Specifically, GlycoBlueTM Coprecipitant (Thermo Fisher,Cat: AM9515) was added in the RNA precipitation step to help visualize the RNA pellet. SMART-Seq V4 Ultra Low Input RNA Kit (Takara, Cat: 634890) was employed for RNA reverse transcription and cDNA amplification. RNA from around 400 cells was loaded in the experiment and 30ng cDNA could be yield after 18 cycles of cDNA amplification. cDNA amount was measured with Qubit dsDNA Quantification Assay kit (High sensitivity) (Thermo Fisher, Cat: Q32851). Quantitative real-time PCR analysis was conducted using TaqMan Probes and TaqMan universal PCR Master Mix (Thermo Fisher, Cat: 4304437). 250pg-500 pg cDNA was loaded for each PCR reaction.

### ATAC-seq

The CX3CR1^+/EGFP^::CCR2^+/RFP^ bone marrow transfer model was used to distinguish periphery infiltrated monocyte and resident microglia. For flow sorting, infiltrated monocytes were gated as CD45^+^CD11b^+^GFP^+^ cells and microglia as CD45^+^CD11b^+^CX3CR1^+^GFP^−^. Each sample was pooled from 5 retinas (from 5 mice) and 1000 to 5000 cells collected and used for ATAC-seq analysis with the ATAC-seq kit from Active motif (Cat: 13150). Briefly, nuclei were isolated by adding 100 μL ice cold ATAC-lysis buffer and then incubated with the tagmentation master mix in a shaking heat block at 37°C/800 rpm for 30 min. Obtained DNA was purified and library generated by PCR reaction for 13 cycles using indexed primers according to the manufacturer’s instructions. A quality control (QC) was performed to verify the size distribution of the PCR enriched library fragments. ATAC-seq sequencing was performed on an Illumina HiSeq 2000 instrument, resulting in 30 million paired-end 50 bp reads per sample. Reads were mapped to the mm9 reference mouse genome using BWA [48]. Those fragments with both ends unambiguously mapped to the genome that were longer than 100 bp were used for further analysis. Hotspot2 was used to detect significant peaks with FDR cutoff of 0.05 [49]. For the analysis of overlap between peak regions, we used a cutoff of 50% reciprocal overlap between the two compared regions. For the analysis of differential chromatin accessibility between groups of replicate samples, DiffBind R package was used [50]: (https://bioconductor.riken.jp/packages/3.2/bioc/vignettes/DiffBind/inst/doc/DiffBind.pdf). Motif analysis was performed through MEME-CHIP (motif analysis of large nucleotide datasets).

### Flat-Mount Staining and Imaging.

1 day, 7 days and 1.5 months after corneal alkali burn injury, mouse retinas were collected and prepared for staining and flat mount. Eyes were first fixed in 4% paraformaldehyde for 2 hours at room temperature. After dissection, retinas were blocked with blocking buffer for 1 hour at room temperature (PBS containing 5% normal donkey serum, 0.25% Triton-X-100). Antibody information can be found in Table 1. For retinal flat-mount preparations, whole retinas were laid flat after radial relaxing incisions and mounted on slides and cover-slipped.

## Supplementary Material

1

## Figures and Tables

**Table 1.** Antibody information for flow Cytometry and immunostaining.

ANTIBODY	FLUOROPHORE/SECONDARY	BRAND	CLONE	CATLOG NUMBER	DILUTION	APPLICATION
**CD45**	Brilliant Violet 785	Biolegend	30-F11	103149	1 to 100	Flow cytometry
**CD11B**	PerCP-Cyanine 5.5	Biolegend	M1/70	101228	1 to 100	Flow cytometry
**LY6C**	Brilliant Violet 421	Biolegend	HK1.4	128031	1 to 100	Flow cytometry
**CX3CR1**	APC	Biolegend	SA011F11	149008	1 to 100	Flow cytometry
**CX3CR1**	Brilliant Violet 605	Biolegend	SA011F11	149027	1 to 100	Flow cytometry
**FCRLS**	Alexa Fluor 647 donkey anti rat	Dr. Margeta			1 to 200	Flow cytometry
**IBA1**	Alexa Fluor 647 donkey anti rabbit	Wako		019–19741	1 to 300	Immunostaining
**TMEM119**	Alexa Fluor 647 donkey anti guinea pig	Synaptic systems		400 004	1 to 300	Immunostaining
**P2RY12**	Alexa Fluor 647 donkey anti rabbit	Dr. Margeta			1 to 200	Immunostaining
**P2RY12**	Alexa Fluor 647 donkey anti rabbit	Anaspec		as-55043a	1 to 200	Immunostaining
**MHC-II**	Alexa Fluor 647 donkey anti rat	BD Bioscience	I-A/I-E	556999	1 to 100	Immunostaining

## References

[1] Kamimura D., , The gateway theory: bridging neural and immune interactions in the CNS. Front Neurosci, 2013. 7: p. 204. 24194696 10.3389/fnins.2013.00204 PMC3810779

[2] Paschalis E.I., , Permanent neuroglial remodeling of the retina following infiltration of CSF1R inhibition-resistant peripheral monocytes. Proc Natl Acad Sci U S A, 2018. 115(48): p. E11359–e11368. 30442669 10.1073/pnas.1807123115 PMC6275537

[3] Paschalis E.I., , The Role of Microglia and Peripheral Monocytes in Retinal Damage after Corneal Chemical Injury. Am J Pathol, 2018. 188(7): p. 1580–1596. 29630857 10.1016/j.ajpath.2018.03.005 PMC6136091

[4] Paschalis E.I., , Microglia Regulate Neuroglia Remodeling in Various Ocular and Retinal Injuries. J Immunol, 2019. 202(2): p. 539–549. 30541880 10.4049/jimmunol.1800982 PMC6325007

[5] Spiteri A.G., , Microglia and monocytes in inflammatory CNS disease: integrating phenotype and function. Acta Neuropathol, 2022. 143(2): p. 179–224. 34853891 10.1007/s00401-021-02384-2 PMC8742818

[6] Butovsky O., , Identification of a unique TGF-β-dependent molecular and functional signature in microglia. Nat Neurosci, 2014. 17(1): p. 131–43. 24316888 10.1038/nn.3599 PMC4066672

[7] Bennett M.L., , New tools for studying microglia in the mouse and human CNS. Proc Natl Acad Sci U S A, 2016. 113(12): p. E1738–46. 26884166 10.1073/pnas.1525528113 PMC4812770

[8] Ronning K.E., Karlen S.J., and Burns M.E., Structural and functional distinctions of co-resident microglia and monocyte-derived macrophages after retinal degeneration. J Neuroinflammation, 2022. 19(1): p. 299. 36510226 10.1186/s12974-022-02652-2 PMC9743742

[9] Paschalis E.I., , Mechanisms of Retinal Damage after Ocular Alkali Burns. Am J Pathol, 2017. 187(6): p. 1327–1342. 28412300 10.1016/j.ajpath.2017.02.005 PMC5455067

[10] Chen X., , Glaucoma after Ocular Surgery or Trauma: The Role of Infiltrating Monocytes and Their Response to Cytokine Inhibitors. Am J Pathol, 2020. 190(10): p. 2056–2066. 32693061 10.1016/j.ajpath.2020.07.006 PMC7527856

[11] Galatro T.F., , Transcriptomic analysis of purified human cortical microglia reveals age-associated changes. Nature Neuroscience, 2017. 20(8): p. 1162–1171. 28671693 10.1038/nn.4597

[12] Gibbings S.L., , Three Unique Interstitial Macrophages in the Murine Lung at Steady State. American Journal of Respiratory Cell and Molecular Biology, 2017. 57(1): p. 66–76. 28257233 10.1165/rcmb.2016-0361OC PMC5516280

[13] Fengyang L., , Single-cell RNA-seq reveals a dynamic shift of engrafted peripheral macrophages in the CNS towards a microglia signature. 2021. 62(8): p. 918–918.

[14] Lei F., , CSF1R inhibition by a small-molecule inhibitor is not microglia specific; affecting hematopoiesis and the function of macrophages. Proc Natl Acad Sci U S A, 2020. 117(38): p. 23336–23338. 32900927 10.1073/pnas.1922788117 PMC7519218

[15] Saeed S., , Epigenetic programming of monocyte-to-macrophage differentiation and trained innate immunity. Science, 2014. 345(6204): p. 1251086. 25258085 10.1126/science.1251086 PMC4242194

[16] Gibbings S.L., , Transcriptome analysis highlights the conserved difference between embryonic and postnatal-derived alveolar macrophages. Blood, 2015. 126(11): p. 1357–66. 26232173 10.1182/blood-2015-01-624809 PMC4566811

[17] van de Laar L., , Yolk Sac Macrophages, Fetal Liver, and Adult Monocytes Can Colonize an Empty Niche and Develop into Functional Tissue-Resident Macrophages. Immunity, 2016. 44(4): p. 755–68. 26992565 10.1016/j.immuni.2016.02.017

[18] Gosselin D., , An environment-dependent transcriptional network specifies human microglia identity. Science, 2017. 356(6344). 10.1126/science.aal3222 PMC5858585 28546318

[19] Dolan M.J., , Exposure of iPSC-derived human microglia to brain substrates enables the generation and manipulation of diverse transcriptional states in vitro. Nat Immunol, 2023. 24(8): p. 1382–1390. 37500887 10.1038/s41590-023-01558-2 PMC10382323

[20] Rowan S., , Involvement of a gut-retina axis in protection against dietary glycemia-induced age-related macular degeneration. Proc Natl Acad Sci U S A, 2017. 114(22): p. E4472–E4481. 28507131 10.1073/pnas.1702302114 PMC5465926

[21] Wolf S.A., Boddeke H.W., and Kettenmann H., Microglia in Physiology and Disease. Annu Rev Physiol, 2017. 79: p. 619–643. 27959620 10.1146/annurev-physiol-022516-034406

[22] Benmamar-Badel A., Owens T., and Wlodarczyk A., Protective Microglial Subset in Development, Aging, and Disease: Lessons From Transcriptomic Studies. Front Immunol, 2020. 11: p. 430. 32318054 10.3389/fimmu.2020.00430 PMC7147523

[23] Zeng J., , The mechanism of microglia-mediated immune inflammation in ischemic stroke and the role of natural botanical components in regulating microglia: A review. Front Immunol, 2022. 13: p. 1047550. 36818470 10.3389/fimmu.2022.1047550 PMC9933144

[24] Choi I., , Autophagy enables microglia to engage amyloid plaques and prevents microglial senescence. Nat Cell Biol, 2023. 25(7): p. 963–974. 37231161 10.1038/s41556-023-01158-0 PMC10950302

[25] Lv Q.K., , Role of alpha-synuclein in microglia: autophagy and phagocytosis balance neuroinflammation in Parkinson’s disease. Inflamm Res, 2023. 72(3): p. 443–462. 36598534 10.1007/s00011-022-01676-x

[26] Zhou C., , Sustained Inhibition of VEGF and TNF-α Achieves Multi-Ocular Protection and Prevents Formation of Blood Vessels after Severe Ocular Trauma. Pharmaceutics, 2023. 15(8). 10.3390/pharmaceutics15082059 PMC10458495 37631272

[27] Satoh J., , TMEM119 marks a subset of microglia in the human brain. Neuropathology, 2016. 36(1): p. 39–49. 26250788 10.1111/neup.12235

[28] Walker D.G., , Patterns of Expression of Purinergic Receptor P2RY12, a Putative Marker for Non-Activated Microglia, in Aged and Alzheimer’s Disease Brains. Int J Mol Sci, 2020. 21(2). 10.3390/ijms21020678 PMC7014248 31968618

[29] Vankriekelsvenne E., , Transmembrane protein 119 is neither a specific nor a reliable marker for microglia. Glia, 2022. 70(6): p. 1170–1190. 35246882 10.1002/glia.24164

[30] Vanneste D., , MafB-restricted local monocyte proliferation precedes lung interstitial macrophage differentiation. Nat Immunol, 2023. 24(5): p. 827–840. 36928411 10.1038/s41590-023-01468-3 PMC10154211

[31] Kenkhuis B., , Co-expression patterns of microglia markers Iba1, TMEM119 and P2RY12 in Alzheimer’s disease. Neurobiol Dis, 2022. 167: p. 105684. 35247551 10.1016/j.nbd.2022.105684

[32] Sideris-Lampretsas G., , Galectin-3 activates spinal microglia to induce inflammatory nociception in wild type but not in mice modelling Alzheimer’s disease. Nat Commun, 2023. 14(1): p. 3579. 37349313 10.1038/s41467-023-39077-1 PMC10287730

[33] Matek W., , Initial experience with the new electronic endoscope. Endoscopy, 1984. 16(1): p. 20–1. 6697977 10.1055/s-2007-1018519

[34] Haimon Z., , Cognate microglia-T cell interactions shape the functional regulatory T cell pool in experimental autoimmune encephalomyelitis pathology. Nat Immunol, 2022. 23(12): p. 1749–1762. 36456736 10.1038/s41590-022-01360-6

[35] Stratoulias V., , ARG1-expressing microglia show a distinct molecular signature and modulate postnatal development and function of the mouse brain. Nat Neurosci, 2023. 26(6): p. 1008–1020. 37169859 10.1038/s41593-023-01326-3 PMC10244174

[36] Wu Q., , Microglial activation and over pruning involved in developmental epilepsy. J Neuropathol Exp Neurol, 2023. 82(2): p. 150–159. 36453895 10.1093/jnen/nlac111

[37] Yin Z., , Identification of a protective microglial state mediated by miR-155 and interferon-gamma signaling in a mouse model of Alzheimer’s disease. Nat Neurosci, 2023. 26(7): p. 1196–1207. 37291336 10.1038/s41593-023-01355-y PMC10619638

[38] Chen X., , Microglia-mediated T cell infiltration drives neurodegeneration in tauopathy. Nature, 2023. 615(7953): p. 668–677. 36890231 10.1038/s41586-023-05788-0 PMC10258627

[39] O’Koren E.G., Mathew R., and Saban D.R., Fate mapping reveals that microglia and recruited monocyte-derived macrophages are definitively distinguishable by phenotype in the retina. Sci Rep, 2016. 6: p. 20636. 26856416 10.1038/srep20636 PMC4746646

[40] Hammond T.R., , Single-Cell RNA Sequencing of Microglia throughout the Mouse Lifespan and in the Injured Brain Reveals Complex Cell-State Changes. Immunity, 2019. 50(1): p. 253–271 e6. 30471926 10.1016/j.immuni.2018.11.004 PMC6655561

[41] Zhou N., , Transcriptional mechanism of IRF8 and PU.1 governs microglial activation in neurodegenerative condition. Protein Cell, 2019. 10(2): p. 87–103. 30484118 10.1007/s13238-018-0599-3 PMC6340890

[42] Fixsen B.R., , SALL1 enforces microglia-specific DNA binding and function of SMADs to establish microglia identity. Nat Immunol, 2023. 24(7): p. 1188–1199. 37322178 10.1038/s41590-023-01528-8 PMC10307637

[43] Kierdorf K., , Bone marrow cell recruitment to the brain in the absence of irradiation or parabiosis bias. PLoS One, 2013. 8(3): p. e58544. 23526995 10.1371/journal.pone.0058544 PMC3592806

[44] Skene P.J., Henikoff J.G., and Henikoff S., Targeted in situ genome-wide profiling with high efficiency for low cell numbers. Nat Protoc, 2018. 13(5): p. 1006–1019. 29651053 10.1038/nprot.2018.015

[45] Dresselhaus E.C. and Meffert M.K., Cellular Specificity of NF-κB Function in the Nervous System. Front Immunol, 2019. 10: p. 1043. 31143184 10.3389/fimmu.2019.01043 PMC6520659

[46] Saban D.R., New concepts in macrophage ontogeny in the adult neural retina. Cell Immunol, 2018. 330: p. 79–85. 29703455 10.1016/j.cellimm.2018.04.008

[47] Yu C., , Microglia versus Monocytes: Distinct Roles in Degenerative Diseases of the Retina. Trends Neurosci, 2020. 43(6): p. 433–449. 32459994 10.1016/j.tins.2020.03.012 PMC7556353

[48] Li H. and Durbin R., Fast and accurate short read alignment with Burrows-Wheeler transform. Bioinformatics, 2009. 25(14): p. 1754–60. 19451168 10.1093/bioinformatics/btp324 PMC2705234

[49] John S., , Chromatin accessibility pre-determines glucocorticoid receptor binding patterns. Nat Genet, 2011. 43(3): p. 264–8. 21258342 10.1038/ng.759 PMC6386452

[50] Ross-Innes C.S., , Differential oestrogen receptor binding is associated with clinical outcome in breast cancer. Nature, 2012. 481(7381): p. 389–93. 22217937 10.1038/nature10730 PMC3272464

