# Supplementary material for: Epigenetic adaptation drives monocyte differentiation into microglia-like cells upon engraftment into the retina
Source: bioRxiv. 2024 Sep 14:2024.09.09.612126. Preprint. [Version 1] doi: 10.1101/2024.09.09.612126 (PMC11419019; doi:10.1101/2024.09.09.612126)

bioRxiv preprint doi: https://doi.org/10.1101/2024.09.09.612126; this version posted September 14, 2024. The copyright holder for this preprint (which was not certified by peer review) is the author/funder, who has granted bioRxiv a license to display the preprint in perpetuity. It is made available under aCC-BY 4.0 International license.

Sup. Figure S1. mRNA expression of P2ry12, TMEM119, FCRLS, and IBA1 by blood monocytes, embryonic retinal microglia, and engrafted monocytes.

The $CX3CR1^{+/GFP}$ bone marrow transfer model was used to distinguish peripheral infiltrating monocytes from resident microglia. Naïve microglia cells and blood monocytes were collected from uninjured bone marrow transferred mice. Injured microglia and engrafted monocytes were collected from retinas 45 days after ocular injury in bone marrow transferred mice. Cells were collected using flow cytometry sorting with a gating strategy as follows: microglia: $CD45^{+}$ $CD11b^{+}GFP^{-negative}CX3CR1/BV605^{+positive}$ or $CD45^{+}$ $CD11b^{+}$ $GFP^{-negative}CX3CR1/APC^{+positive}$, blood monocyte and engrafted monocytes: $CD45^{+}$ $CD11b^{+}$ $GFP^{+positive}CX3CR1/BV605^{+positive}$ or $GFP^{+positive}CX3CR1/APC^{+positive}$. RNA was isolated and mRNA expression of P2ry12, TMEM119, FCRLS and IBA1 genes was evaluated by PCR. P2ry12, TMEM119, and IBA1 mRNA level were normalized to blood monocyte, while the FCRLS mRNA level was normalized to naïve microglia. No FCRLS was detected in blood monocytes. This data indicates P2ry12, TMEM119, FCRLS, and IBA1 mRNA in engrafted monocytes is increased after retinal engraftment compared to blood monocytes.

Sup. Figure S2. Circulating monocytes do not express microglia makers.

Immunostaining of circulating blood monocytes from $CX3CR1^{+/GFP}$::$CCR2^{+/RFP}$ transgenic mice shows absence of P2ry12, IBA1, and TMEM119 expression. MHC II staining is present in engrafted monocytes. *Scare bar = 20 μm*

Sup. Figure S3. P2ry12 expression in engrafted monocyte at 14 days.

The $CX3CR1^{+/GFP}$::$CCR2^{+/RFP}$ bone marrow chimera model was employed to differentiate engrafted monocytes ($GFP^{+}CCR2^{+}$) from embryonic microglia (GFP^{-} $CCR2^{-}$). Peripheral monocytes acquired P2ry12 expression 14 days after engraftment into the retina. *Scale bar = 50 μm.*

bioRxiv preprint doi: https://doi.org/10.1101/2024.09.09.612126; this version posted September 14, 2024. The copyright holder for this preprint (which was not certified by peer review) is the author/funder, who has granted bioRxiv a license to display the preprint in perpetuity. It is made available under aCC-BY 4.0 International license.

Sup. Figure S4. Protein expression of Iba1 in engrafted monocytes.

The CX3CR1$^{+/GFP}$::CCR2$^{+/RFP}$ bone marrow chimera model was employed to differentiate engrafted monocytes from embryonic microglia. Eighty-five percent of engrafted GFP^{+} monocytes become IBA1^{+} at day 1 of infiltration, while all become IBA1^{+} at 7 and 45 days post engraftment. *ns: Not significant. Yellow arrows indicate GFP$^{-negative}$ IBA1$^{+positive}$ microglia cells and white arrows GFP$^{+positive}$ engrafted monocytes. Scare bar = 50 µm.*

Sup. Figure S1

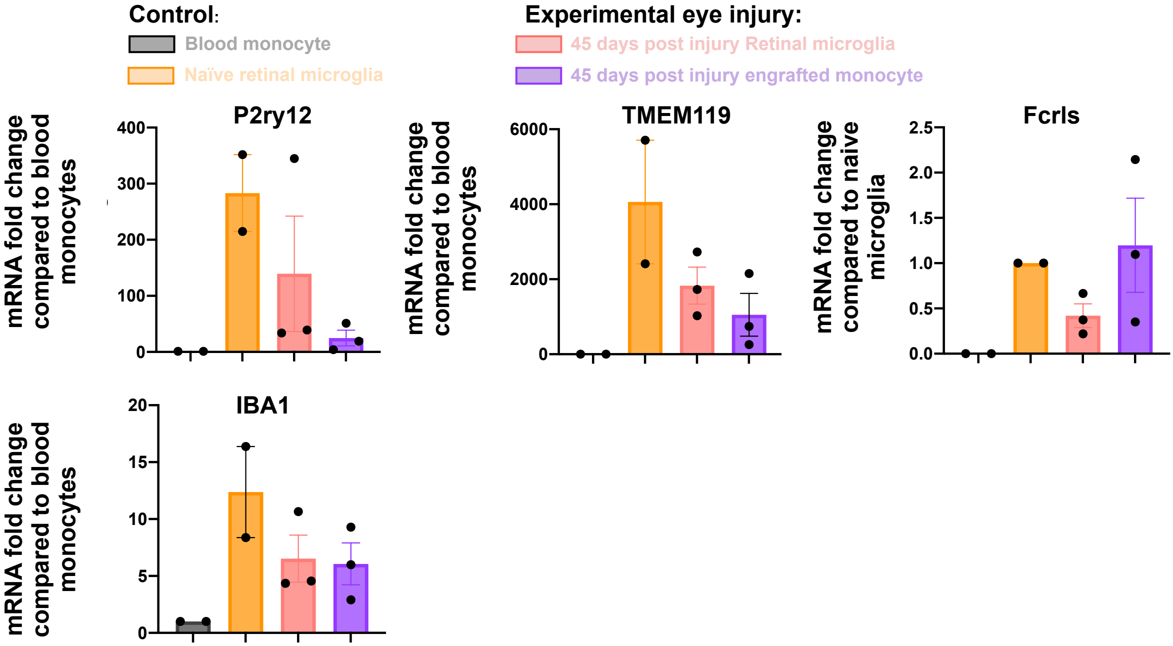

Sup. Figure S2

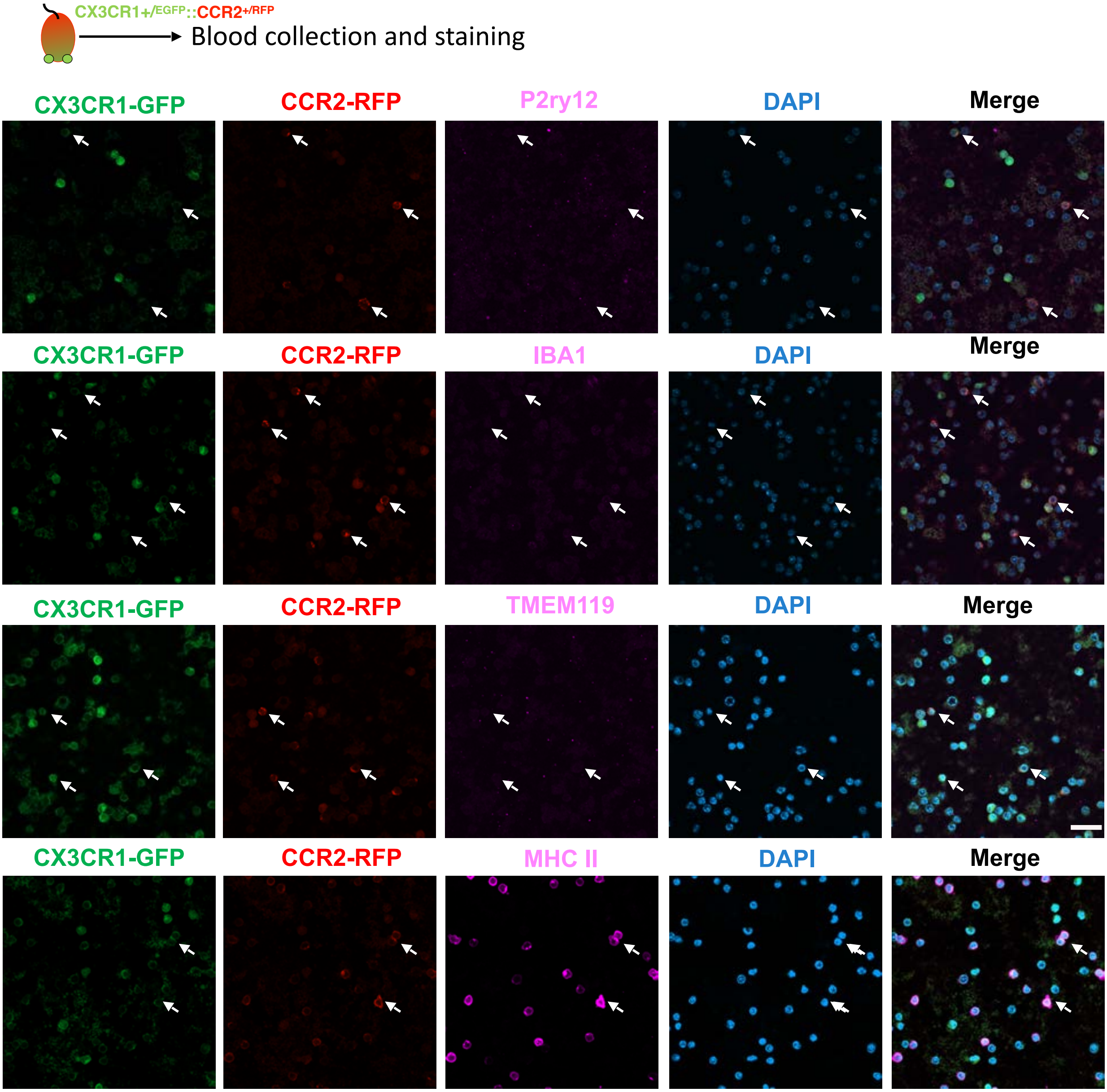

Sup. Figure S3

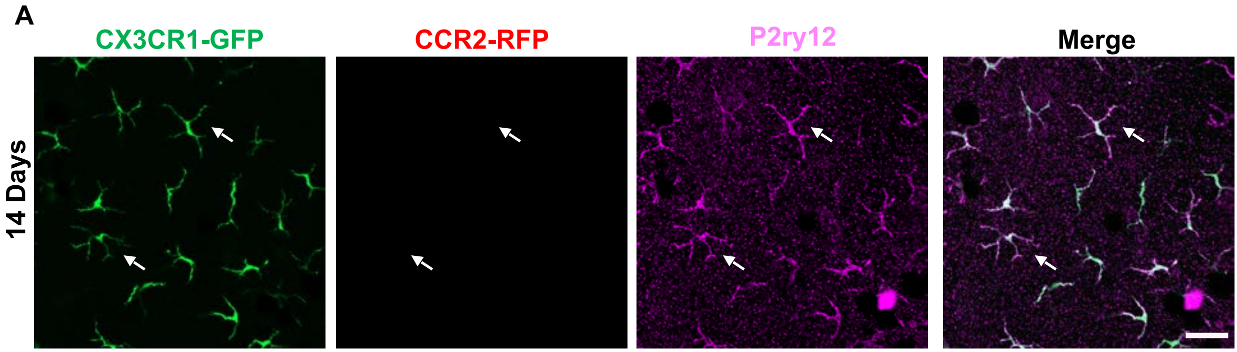

Sup. Figure S4

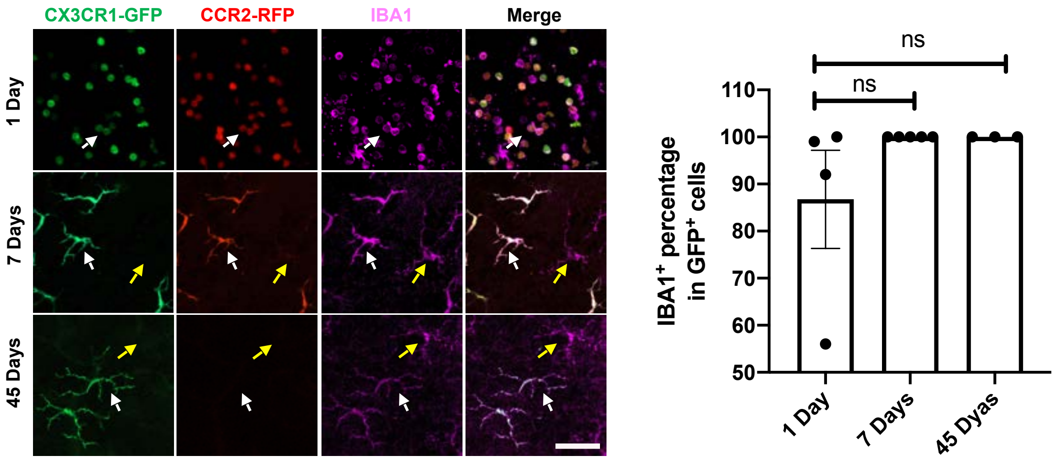

Supplement: 1 [file NIHPP2024.09.09.612126V1-supplement-1.pdf]
